# Evaluation of the Cathodic Electrodeposition Effectiveness of the Hydroxyapatite Layer Used in Surface Modification of Ti6Al4V-Based Biomaterials

**DOI:** 10.3390/ma15196925

**Published:** 2022-10-06

**Authors:** Michalina Ehlert, Aleksandra Radtke, Michał Bartmański, Piotr Piszczek

**Affiliations:** 1 Department of Inorganic and Coordination Chemistry, Faculty of Chemistry, Nicolaus Copernicus University in Toruń, Gagarina 7, 87-100 Toruń, Poland; 2 Nano-Implant Ltd., Gagarina 7/47, 87-100 Toruń, Poland; 3 Department of Biomaterials Technology, Faculty of Mechanical Engineering and Ship Technology, Gdańsk University of Technology, Gabriela Narutowicza 11/12, 80-233 Gdańsk, Poland

**Keywords:** cathodic electrodeposition, hydroxyapatite, nanomechanical properties, surface modification, Ti6Al4V alloy

## Abstract

The important issue associated with the design and the fabrication of the titanium and titanium alloy implants is the increase of their biointegration with bone tissue. In the presented paper, the research results concerning the conditions used in the cathodic deposition of hydroxyapatite on the surface Ti6Al4V substrates primarily modified by the production of TiO_2_ nanoporous coatings, TiO_2_ nanofibers, and titanate coatings, are discussed. Despite excellent biocompatibility with natural bone tissue of materials based on hydroxyapatite (HA), their poor adhesion to the substrate caused the limited use in the implants’ construction. In our works, we have focused on the comparison of the structure, physicochemical, and mechanical properties of coating systems produced at different conditions. For this purpose, scanning electron microscopy images, chemical composition, X-ray diffraction patterns, infrared spectroscopy, wettability, and mechanical properties are analyzed. Our investigations proved that the intermediate titanium oxide coatings presence significantly increases the adhesion between the hydroxyapatite layer and the Ti6Al4V substrate, thus solving the temporary delamination problems of the HA layer.

## 1. Introduction

Titanium and its alloys are wieldy used as load-bearing implants [1,2,3]. Among them, the Ti6Al4V alloy in particular has been considered as one of the suitable candidates for bioimplants due its good biocompatibility, well-balanced strength-to-weight ratio and comparatively low elastic modulus [4,5,6]. Nevertheless, research is still being conducted to achieve a higher biointegrity of the Ti6Al4V alloy and closer mechanical properties to the skeletal system. It should also be noted that the studies of titanium-based materials used in the construction of modern implants revealed their susceptibility to bacteria-induced inflammations [7,8,9]. It can lead to the loosening of the implants and other complications and consequently, their removal or replacement may be necessary. The surface modification of implants by producing the coatings of defined architecture, which will inhibit the adhesion of bacterial cells to them, is one of the proposed solutions. Electrolytic oxidation (anodizing), allowing for the production of different surface architecture coatings, i.e., nanoporous, nanotubular, or nanosponge-like types, is a widely used method [10,11,12]. Another good example is the result of investigations concerning the Ti6Al4V implant surface modification using SLS technology (selective laser sintering). It was found that Ti6Al4V implant surface modification by the TiO_2_ nanoporous layer covering, led to the formation of a system of more suitable physicochemical and osseointegration properties [10,11,13]. Moreover, this layer exhibited excellent wear resistance and the ability to propagate energy at plastic deformation during loading. The increase in the antimicrobial activity of this implant was obtained by surface enrichment with metallic silver nanoparticles. A significant advantage of the modification type mentioned above was that the layer did not release substances showing mutagenic properties [14,15]. 

From the new generation implant construction point of view, the crucial issue is to equip their surface with a biocompatible, bioactive coating layer that adheres perfectly to the substrate (e.g., does not delaminate during use of the implant) [16,17]. The way to achieve this effect can be a production of an additional coating of a bone-like substance that is hydroxyapatite (HA), i.e., hydroxyl-containing forms of calcium phosphate, which promotes bone formation and growth [17,18]. Crystalline HA provides mechanical stability to the coating. However, in the environment of body fluid it can slowly degrade, leading to insufficient bone ingrowth [19]. On the other hand, the amorphous type has a slightly higher solubility, which promotes faster initial bone fixation due to resorption and bioactivity [20]. For biomedical applications, an important ceramic material is a hydroxyapatite with a Ca/P molar ratio of 1.67. Since it can provide the mineral scaffolding for connective tissue, which is responsible for the mechanical strength of bones [16,21]. The use of hydroxyapatite as a protective coating for various metallic implants offers many advantages. The surfaces of metal implants become more resistant to corrosion and are also protected against releasing toxic ions from their surfaces [21]. Furthermore, the production of a hydroxyapatite coating promotes the formation of apatite, the main inorganic component of bone and teeth [22,23,24]. However, calcium phosphate coatings often exhibit poor adhesion to metallic substrates. Implants with an HA layer may undergo fatigue failure in load-bearing applications. According to literature data, it is assumed that in order to avoid fatigue damage and dissolution, the thickness of the HA layer should be ca. 50 μm [22,25].

The plasma spraying, pulsed laser deposition, sol-gel method, PVD (sputter coat-ing), electrophoretic deposition, electrodeposition, and gas detonation deposition are usually used for producing an HA layer on implant surfaces [16,17,21,24,26,27,28]. Plasma spraying is the most popular, which has the advantage of being able to produce highly repeatable coatings. Nevertheless, the plasma spraying process uses high temperatures, often linked to uncontrolled phase changes and thermomechanical mismatches [16,27,29,30]. An alternative method to produce hydroxyapatite layers is electrodeposition. This is an efficient technique for producing a calcium phosphate layer on the surface of a conductive material through a series of chemical reactions in an aqueous solution using electrical energy [16,21,24,27,31]. The electrochemical technique is attractive due to its simplicity, low cost, uniformity of the produced coatings, and ability to precisely control the properties of the layers produced (chemical composition, thickness, structure) at low temperatures. It is suitable for coating surfaces with complex geometries [16,17,21,32]. The major limitation of the electrodeposition technique is the poor adhesion of the HA layer to the implant’s surface [16,21,33]. Usually, the interfacial delamination of the HA layer is initially caused by vertical cracking of the coating throughout the thickness, which occurs at the HA–Ti6Al4V and HA–human bone interface [34]. The delamination of the hydroxyapatite layer can lead to the formation of inflammation in the surrounding organs, and consequently bone loss and even loosening of such an implant [22,35]. The analysis of literature data shows that research on this issue is conducted in various directions. An example is using computational models to the analyze Tresca stresses in metal bearings, as well as to assess delamination-fretting damage [34,36]. According to Ammarullah et al., it is the Ti6Al4V-on-Ti6Al4V bearings that show the superior Tresca stress reduction properties among other metal-on-metal bearings [36]. 

Nevertheless, so far, research using a connector has not yielded the expected results, so we aimed to produce a group of different connectors and select the most optimal one to create more stable HA layers [22,37]. For this purpose, a multistage chemical treatment of the Ti6Al4V substrate surface was used to create a stable intermediate layer connecting the alloy surface with the hydroxyapatite layer. Based on our earlier works, the following coatings were selected: (a) nanoporous TiO_2_ coatings, (b) nanofibrous TiO_2_ coatings, and (c) titanate coatings. It should be noted that we tested the coatings mentioned above as independent systems but not as intermediate ones [10,38,39]. For this reason, an innovative aspect of the presented paper is the system’s production: Ti6Al4V–intermediate layer (TiO_2_ or titanate)–HA. Titanium oxide coatings show high biointegration properties and better mechanical properties than titanium alloy. However, their Young’s modulus and hardness parameters are still higher than cortical bone’s. Therefore, our research aimed to produce a coating system whose mechanical parameters are like the cortical bone and reveal the hydroxyapatite layer’s high adhesion strength to the Ti6Al4V substrate. To achieve this effect, we optimized the process conditions of the cathodic electrodeposition of hydroxyapatite and the determined mechanical properties of produced systems Ti6Al4V-intermediate layer-HA.

## 2. Materials and Methods

### 2.1. The Synthesis of TiO_2_ and Titanate Nanocoatings

All experiments were carried out using Ti6Al4V alloy (marked as T, grade 5, 99.7% purity) (Strem Chemicals Inc., Bischheim, France), a foil of 0.2 mm thickness, as a base substrate, which was cut into strips of 6 mm wide and 100 mm long. In the first stage, the surface of T substrates was modified by manufacturing TiO_2_ nanoporous (T5) coatings and TiO_2_ nanofiber (TNF6 and TNF72), and titanate (T5-S and T-S) types, according to methods previously described (all acronyms used in this article are consistent with those used to mark samples in earlier works) [10,11,38,39,40]. Before the next stages of the research, the samples were structurally and morphologically characterized.

Details of T surface modification:

(a) The anodic oxidation method allowed the production of T5 coatings on the surface of Ti6Al4V substrates. This method used the 0.3% HF solution as an electrolyte, t = 20 min, and the 5 V potential. The produced T5 coatings were rinsed with deionized water and in acetone for 10 min.
(b) The TNF coatings were produced due to T sample surface chemical oxidation. The surfaces of the substrates were chemically etched in c.a. 5.8 M HCl. Afterwards, materials were heated in 30% H_2_O_2_ solution at 358 K under a reflux condenser (TNF6C) or in an incubator (TNF6S) for t = 6 h. The nanofibrous samples marked TNF72 were produced in a slightly different procedure. The surface etching of the Ti6Al4V substrate was applied (a) in 2M HF solution for 10 s (TNF72a) or (b) in a 1:4:5 mixture of HF:HNO_3_:H_2_O (TNF72b). Then, the samples were immersed in 30% H_2_O_2_ solution at 358 K for 72 h in an incubator.
(c) The alkali-sodium treatment produced the T-S and T5-S samples. The Ti6Al4V sample (T) and nonporous one (T5) were immersed in a 7 M NaOH solution at 339 K for t = 48 h. In the next step, these materials were washed with distilled water and dried at 314 K for 24 h in an incubator.

### 2.2. Synthesis of Hydroxyapatite on TiO_2_ and Titanate Nanocoatings 

**Figure 1:**
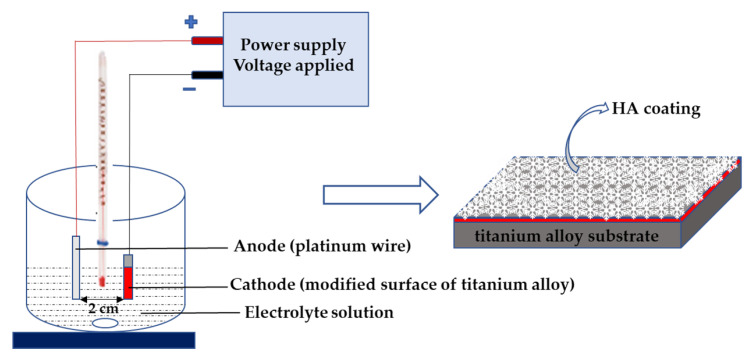
Scheme of hydroxyapatite coatings fabricated by electrodeposition.

The electrochemical cathodic deposition method was applied when depositing the hydroxyapatite (HA) coating. The experimental set-up is home-made and was made by the authors of this paper. It consists of a laboratory power supply (China, MCP lab electronics, M10-QS1005; display accuracy is ± 0.5%Rdg + 2digits) and two digital multimeters (Germany, Beha Amprobe, AM-510-EUR; accuracy of DC current measurement is ± 1%Rdg + 2digits; accuracy of DC voltage measurement is ± 1%Rdg + 3digits). The deposition process was carried out at 339 K temperature using prepared substrate ((T (Ti6Al4V), TiO_2_ nanoporous (T5), titanate (T5-S, T-S), and TiO_2_ nanofibres (TNF6C, TNF6S, TNF72a, and TNF72b)) as cathode, platinum wire as anode (Figure 1). The electrolyte consisted of Ca(NO_3_)_2_ · 4 H_2_O (0.042 M), NH_4_H_2_PO_4_ (0.025 M), and EDTA-2Na (1.5 × 10^−3^ M) in distilled water. The initial pH value of the electrolytes was adjusted to 4.5 using Tris(hydroxymethyl)aminomethane. The deposition process time was 60 min. The electrochemical deposition of HA on each sample was carried out at three different current values, i.e., 1.5 mA, 2.5 mA, and 3.5 mA. The voltage range during deposition was 2.24−3.00 V. After cathodic deposition, specimens were immersed in 0.1M NaOH solution for 2 h at 339 K and then sintered at 524 K for 2 h. All samples prepared for analyses were autoclaved using an IS YESON YS-18L (Yeson, Ningbo, China) at 396 K, p = 120 kPa, t = 20 min. 

### 2.3. Structural and Morphological Characterization

The surface morphology of all samples was studied using a Quanta scanning electron microscope with field emission (SEM, Quanta 3D FEG, Huston, TX, USA). The phase identification of all produced layers was carried out with X-ray diffraction (XRD; PANalytical X’Pert Pro, PANalytical B.V., Almelo, the Netherlands; MPD X-ray diffractometer using Cu-K alfa radiation, grazing incidence angle mode–GIXRD; the incidence angle was equal to 1 degree). Diffuse reflectance infrared Fourier transform spectra (DRIFT, Spectrum 2000, PerkinElmer Inc., Waltham, MA, USA) were used to estimate the structure of the specimens. The chemical composition of all synthesized layers was determined using an energy-dispersive X-ray spectrometer (EDS, Quantax 200 XFlash 4010, Bruker AXS, Karlsruhe, Germany). Surface roughness were carried out using a commercial Nanoscope IIIa MultiMode AFM (Veeco Metrology Inc., Santa Barbara, CA, USA) through the technique of tapping mode, an area of 10 × 10 × 2.5 µm. All analyses were conducted for five samples of each modification.

### 2.4. Contact Angle

The contact angle of water on all samples produced at different currents was measured at room temperature using a goniometer (DSA 10 Krüss GmbH, Hamburg, Germany) with drop shape analysis software (ADVANCE, Krüss software, Krüss GmbH, Hamburg, Germany). The volume of the deionized water drop in the contact angle measurement was 3 mL for onto each sample. The contact angle was measured in triplicate and the mean value was calculated.

### 2.5. Nanomechanical Properties and Adhesion

The nanomechanical properties, hardness, and Young’s modulus were studied using the nanoindentation technique with Oliver–Pharr procedure with nanoindenter (NanoTest Vantage, Wrexham, United Kingdom) and Berkovich indenter. The 25 independent indentations were performed on all tested materials on three samples of each group (n = 3). The increased to maximum load was 50 mN and the time was 10 s, with 5 s dwell with maximum load and unloading to zero force equal to 10 s. After indentation, a temperature drift was performed at a load of 5 mN for 30 s. The distance between indentations was 20 µm on both axes. To convert the reduced Young’s modulus for Young’s modulus, according to the Oliver–Pharr procedure, the Poisson’s modification ratio of 0.28 was used. The adhesion of modifications to titanium substrate was studied with a nanoscratch-test. The nanoscratch-test was performed using a nanoindenter (NanoTest Vantage, Wrexham, The United Kingdom) and a diamond indenter. Ten measurements on three samples of each group (n = 3) were made for each sample tested with a maximum force of 500 mN and over a length of 500 µm. The distance between measurements was 250 µm. The adhesion force of the modification to the titanium substrate was evaluated as a sudden and abrupt change in the plot of the normal force against the friction force recorded during the measurement.

## 3. Results

### 3.1. Structural and Morphological Characterization of TiO_2_ and Titanate Nanocoatings

**Figure 2:**
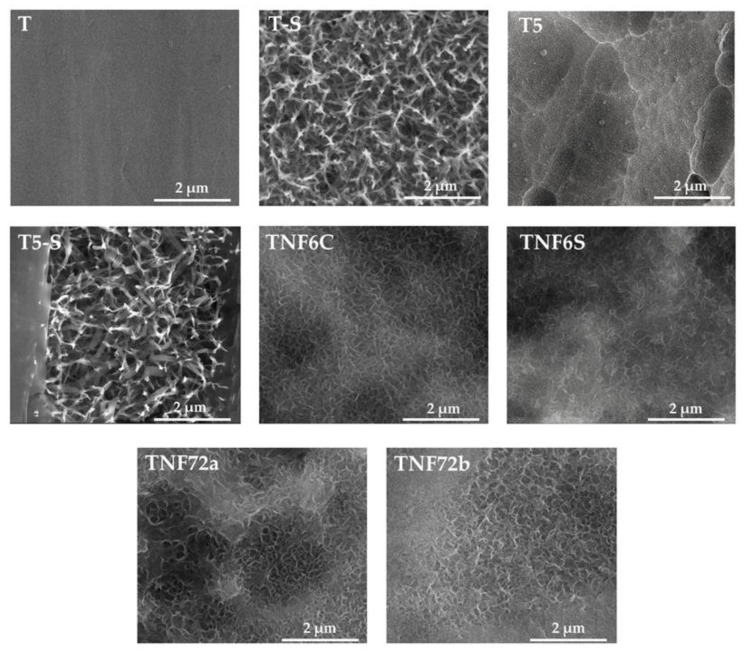
SEM images of T, T-S, T5, T5-S, TNF6C, TNF6S, TNF72a, and TNF72b surface sample morphologies.

In order to improve the physicochemical properties of Ti6Al4V samples (T), their surface was modified by the production of TiO_2_-based coatings, which showed suitable mechanical and physicochemical parameters and high biointegration activity using the previously described methods [10,11,38,39,40]. The morphological differences between T, T-S, T5, T5-S, TNF6C, TNF6S, TNF72a, and TNF72b samples are presented in Figure 2.

Searching for the most optimal and repeatable HA synthesis procedure, which can be applied to biomaterials with different nanoarchitectures, our research started by determining such parameters of cathodic deposition as temperature (T) and process time (*t*) and the effect of EDTA-2Na addition to the electrolyte. In the carried out experiments, the T was changed between 339 and 364 K, *t* = 25 − 60 min and we tested the use of the electrolyte solution with and without the addition of 1.5 × 10^−3^ M EDTA-2Na. The results revealed that the following conditions were the best—electrolyte containing the EDTA-2Na, T = 339 K, and *t* = 60 min.

**Figure 3:**
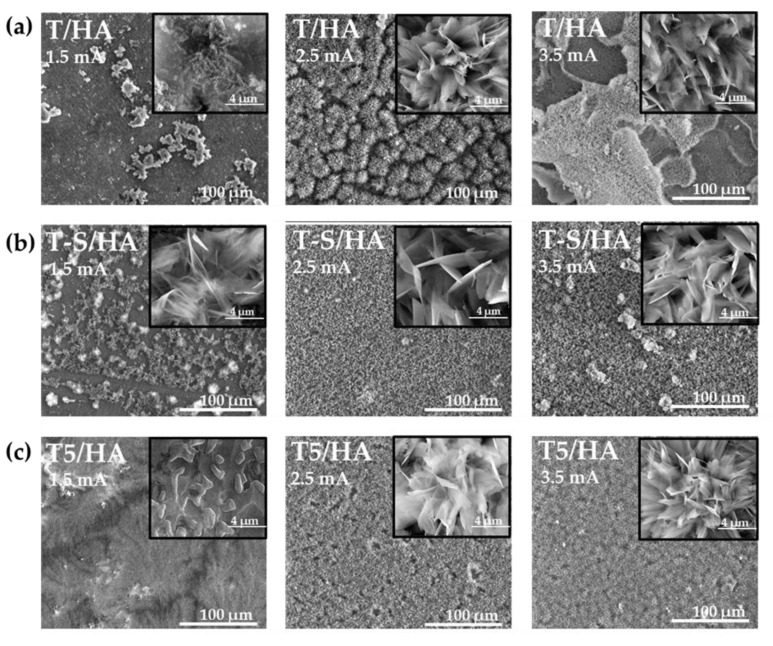

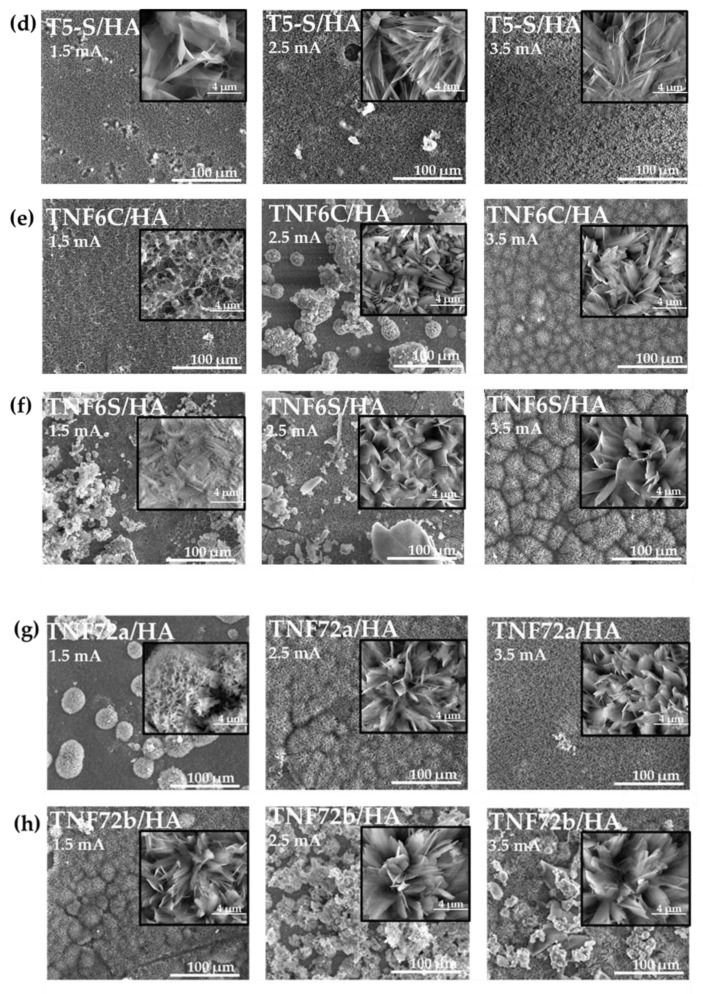
SEM images of the surface morphology of the T/HA (**a**), T-S/HA (**b**), T5/HA (**c**), T5-S/HA (**d**), TNF6C/HA (**e**), TNF6S/HA (**f**), TNF72a/HA (**g**), and TNF72b/HA (**h**) samples obtained at various currents (1.5, 2.5, 3.5 mA).

The differences in surface morphology of samples T/HA (a), T-S/HA (b), T5/HA (c), T5-S/HA (d), TNF6C/HA (e), TNF6S/HA (f), TNF72a/HA (g), and TNF72b/HA (h), which were produced at various currents (1.5 mA; 2.5 mA; 3.5 mA) are presented in Figure 3. The morphology and packing density of hydroxyapatite layers significantly depended on the type of substrate and on the current density applied during cathodic deposition. For T/HA, T5/HA, TNF6C/HA, and TNF6S/HA substrates, the applied current of 1.5 mA was too low, and the dispersed HA nucleations of different morphology were formed (Figure 3a,c,e,f)). In the case of T-S/HA, T5-S/HA, TNF72b/HA samples, the hydroxyapatite was formed in the shape of nanoplates (Figure 3b,d,h), while for TNF72a/HA, a floral morphology composed of thin nanoplates was formed (Figure 3g). By increasing the current intensity and thus the current density for all substrates, the hydroxyapatite morphology changed to a floral morphology composed of numerous nanoplatelets. Furthermore, with increasing current density, there is a change in the hydroxyapatite structure from dispersed to densely packed (Figure 3a–h). The further enhancement of the current density up to 3.5 mA led to the delamination of the hydroxyapatite layer deposited on the surface of nonmodified Ti6Al4V substrates (T/HA, Figure 3a). According to literature reports, the intensity of hydrogen gas escaping in the cathodic reaction increases with increasing current density, which can lead to cracks in the hydroxyapatite layer [33,34]. Figures S1 and S2 show SEM cross-section images of the T/HA (a), T-S/HA (b), T5/HA (c), T5-S/HA (d), TNF6C/HA (e), TNF6S/HA (f), TNF72a/HA (g), and TNF72b/HA (h) samples at 2.5 mA current (Figure S1) or 3.5 mA current (Figure S2). By increasing the current intensity, we observe the formation of thicker hydroxyapatite layers on the surface of all the analyzed biomaterials. The most significant growth of the HA layer was noted for T-S/HA (from ~12.58 µm to ~17.60 µm) and TNF72b (from ~14.51 µm to ~20.80 µm) samples (Figures S1 and S2).

Scanning electron microscopy with energy-dispersive spectroscopy (SEM/EDS) analysis revealed mainly calcium, phosphorous, titanium, vanadium, aluminium, and oxygen to be present in the surface of all biomaterials. Small amounts of sodium were additionally detected on the alkali-modified surfaces (T-S/HA and T5-S/HA). The Ca/P molar ratios for the samples with hydroxyapatite layers at different currents are shown in Table 1. A smaller Ca/P ratio for samples T5/HA (0.82) and TNF6C/HA (1.13) at 1.5 mA may indicate the formation of a calcium-poor layer. On the surface of titanium alloy T, nanoporous T5, as well as after alkali-sodium treatment of T-S and T5-S, the most similar Ca/P ratio calculated directly from the EDS results to stoichiometric hydroxyapatite Ca/P ratio (1.67) was obtained during hydroxyapatite deposition at 2.5 mA [16,21,24,26]. In contrast, for TNF6 and TNF72 nanofibrous coatings, the Ca/P ratio is relatively close to the theoretical value during HA deposition at 3.5 mA. SEM/EDS analysis confirmed the presence of hydroxyapatite on titanium oxide surfaces (Figure S3).

**Figure 4:**
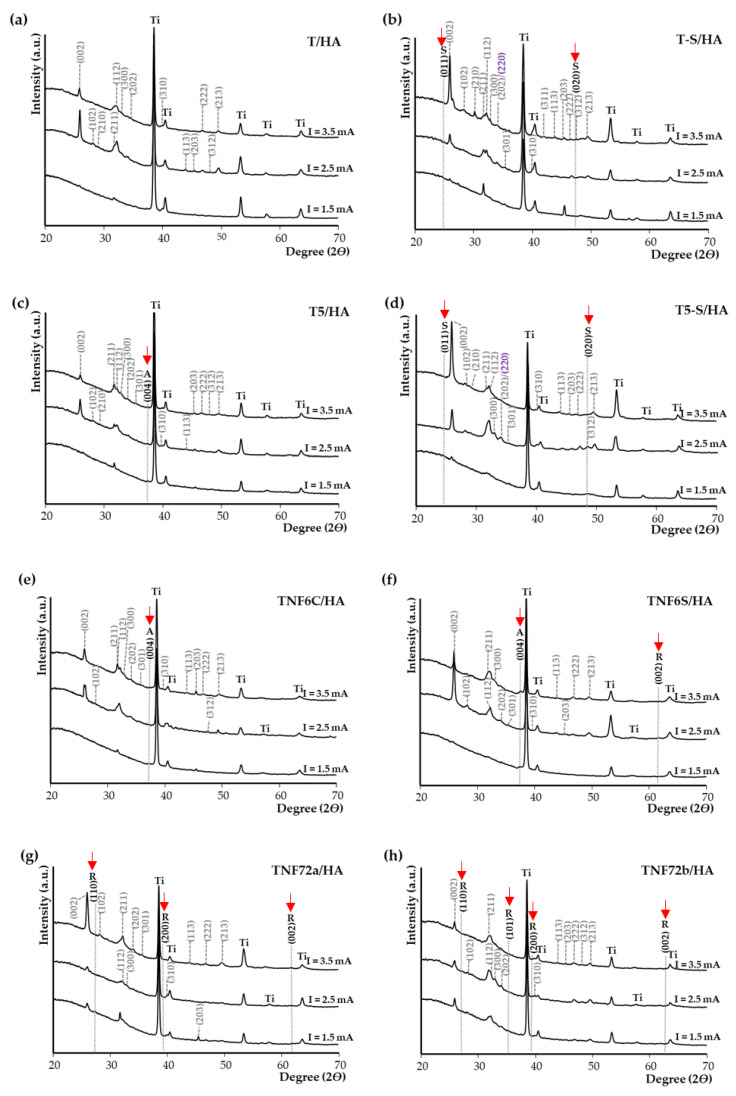
X-ray diffraction patterns of T/HA (**a**), T-S/HA (**b**), T5/HA (**c**), T5-S/HA (**d**), TNF6C/HA (**e**), TNF6S/HA (**f**), TNF72a/HA (**g**), and TNF72b/HA (**h**) samples at various currents (1.5 mA; 2.5 mA; 3.5 mA). (hkl) for HA are marked by grey colour. (hkl) for CaTiO_3_ are marked in violet. S is assigned to the sodium titanate. Ti is assigned to the Ti6Al4V substrate (TiO_2_ anatase phase (A) and rutile phase (R)).

The hydroxyapatite (HA) presence on the surface of samples with different morphology was confirmed by X-ray diffraction (XRD). Figure 4 shows the XRD patterns of T/HA (a), T-S/HA (b), T5/HA (c), T5-S/HA (d), TNF6C/HA (e), TNF6S/HA (f), TNF72a/HA (g), and TNF72b/HA (h) samples at various currents (1.5 mA; 2.5 mA; 3.5 mA). The results demonstrate that a pure layer of hydroxyapatite (Ca_10_(PO_4_)_6_(OH)_2_) was deposited on all analyzed surfaces of biomaterials at 2.5 mA and 3.5 mA. At 1.5 mA a peak of very low intensity is observed, in particular for samples T/HA, T5/HA, TNF6C/HA, and TNF6S/HA. As can be seen, the XRD pattern of the peak at 2θ of ~25.9° corresponding to plane (002) is the strongest among the other peaks in the diffraction patterns. According to literature reports, this may be due to the fact that HA crystals often form in a direction perpendicular to the electrode surface in the electrodeposition method [41,42]. The positions of the HA peaks marked on the spectra are in good agreement with the data in card JCPDS no. 09-0432.

The diffuse reflectance infrared Fourier transform spectra (DRIFT), registered for HA layers produced at different currents, are shown in Figure S4. The detected frequencies and corresponding functional groups in hydroxyapatite samples are listed in Table 2. 

The DRIFT results of all studied samples were consistent with the SEM images and EDS, as well as XRD data. At 1.5 mA, bands attributed to phosphate groups in hydroxyapatite are invisible (TNF6C/HA, TNF72a/HA) or weakly intense (TNF72b/HA) on modified nanofibrous surfaces, except for the TNF6S/HA sample. For this sample, the intense bands assigned to CO_3_^2−^ group in carbonate HA (stretching mode of ν_1_ at 1460 cm^−1^ and 1406 cm^−1^, as well as bending mode of ν_3_ or ν_4_ at 872 cm^−1^) were found [43,44,45,46,47,48,49,51,52]. However, the HPO_4_^2−^ group in the crystal lattice could also be responsible for the additional low-intensity band centered around 875–867 cm^−1^, which indicates the formation of non-stoichiometric HA [43,50,54]. In the case of the other T/HA, T-S/HA, T5/HA, and T5-S/HA samples, at 1.5 mA the appearance of intense bands attributed to PO_4_^3−^ groups was noticed. The additional band at 495 cm^−1^ was detected in the spectrum of T5/HA sample was assigned to Ca-Ti-O group modes. While the bands between 699 and 800 cm^−1^ were attributed to the Ti–O vibration [38,39,45,56,57]. The bands attributed to O-Ti-O modes at 777 cm^−1^ could also be found in the spectra of the TNF6C/HA 2.5 mA samples. The characteristic bands of the hydroxyapatite (PO_4_^2−^, OH-, and sometimes CO_3_^2−^) were found in spectra of all samples produced at 2.5 and 3.5 mA. The sharp band localized at 1594–1653 cm^−1^ and the broad band between 3000 and 3456 cm^−1^ were attributed to the vibrations of the absorbed H_2_O molecules in the HA structure (they were assigned to HOH bending and OH stretching modes, respectively) [10,19,38,39,41,42,43,44,45,46,49,50,51,55,56,58,59]. These bands are least noticed for the T5/HA and T5-S/HA sample’s surface layer.

The surface roughness (R_a_) of the hydroxyapatite layers was calculated from the AFM topographic images, and the mean values in micrometer (µm) are summarized in Table 3. The results demonstrated that, with increasing current intensity (from I = 2.5 mA to I = 3.5 mA), the roughness increases for all tested samples with oxide intermediate. The highest surface roughness (0.61 µm) was obtained for the TNF6C/HA sample (nanofibrous intermediate layer, oxidized for 6 h in hydrogen peroxide solution) at 3.5 mA. In the case of Ti6Al4V/HA sample (without intermediate layer) at I = 3.5 mA, the R_a_ value was significantly lower (0.33 µm). However, for the HA layer deposited at I = 2.5 mA, the R_a_ value increases up to 0.57 µm (Table 3). This may be due to the delamination of the HA layer from the titanium alloy substrate at higher current densities.

### 3.2. Contact Angle 

The water contact angle for all samples with the HA layer, produced at different currents (1.5, 2.5 and 3.5 mA), was nearly 0°. On all the surfaces of the samples with the hydroxyapatite layer, the drop of water spread rapidly, which means that these surfaces show a clear superhydrophilic character.

### 3.3. Mechanical Properties and Adhesion

**Figure 5:**
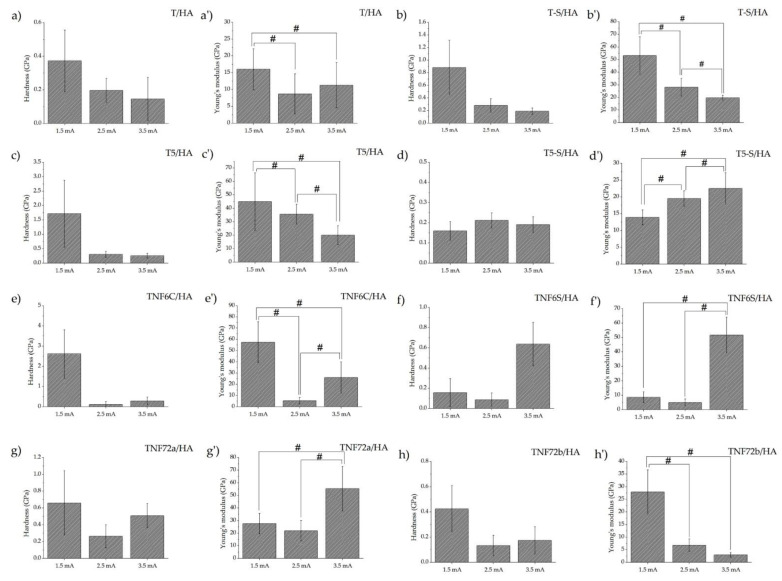
Nanomechanical properties of studied T/HA ((**a**) for hardness and (**a’**) for Young’s modulus), T-S/HA ((**b**,**b’**)), T5/HA ((**c**,**c’**)), T5-S/HA ((**d**,**d’**)), TNF6C/HA ((**e**,**e’**)), TNF6S/HA ((**f**,**f’**)), TNF72a/HA ((**g**,**g’**)), and TNF72b/HA ((**h**,**h’**)) samples at various currents (1.5 mA; 2.5 mA; 3.5 mA); (# significantly different according to one-way ANOVA test followed by Bonferroni’s multiple comparison test, *p* < 0.05).

Figure 5 shows the results of the measurements of nanomechanical properties. The main mechanical properties are hardness and Young’s modulus. Although the instrumental values of these parameters are obtained in the nanoindentation test, they provide an overview and the opportunity to compare these properties. The occurrence of high standard deviations in nanoindentation studies of porous ceramic materials, such as hydroxyapatite coatings, are normal occurrences. In addition, when correlating the mechanical test results with SEM images of the tested specimens, it should be noted that in the case of hydroxyapatite coatings obtained at the lowest current value (1.5 mA), the coatings are characterized by unevenness. The test results presented probably concern either the coating or the substrate material. Figure S5a,b shows the nanohardness and Young’s modulus T-S and T5-S specimens. In the case of T-S and T5-S samples, no effect of modification on the hardness value was observed, only in T5-S did the value of Young’s modulus increased almost twice. For the rest of the tested materials, the results obtained indicate that there is no significant effect of the modification manufacturing parameters on hardness. In the case of Young’s modulus, the obtained results of nanomechanical properties indicate that the influence is significant for most samples. Increasing the deposition current from 1.5 mA to 3.5 mA for all tested specimens had a significant effect on the value of reduced Young’s modulus. An increase in Young’s modulus with increasing deposition current was observed for the T5-S/HA samples, while a decrease in Young’s modulus with increasing current was observed for T-S/HA, T5/HA, and TNF72b/HA. The optimal mechanical properties of the implants for biomechanical compatibility should be as close as possible to human cortical bone (hardness 0.3–0.7 GPa and Young’s modulus ~20 GPa), but it is Young’s modulus that is crucial. In the case of the tested materials, a Young’s modulus close to that of bone, with appropriate hardness, was obtained for TS-HA (2.5 mA), T5/HA (3.5 mA), TNF6C/HA (3.5 mA), and TNF72a/HA (1.5 mA) samples. However, due to the previous description of the HA coating inhomogeneity for a low value of deposition current (1.5mA), TNF72a/HA samples should not be considered. Comparing the results of the nanomechanical tests with previous studies for all the samples tested, the hardness and Young’s modulus results obtained are lower on specimens with the hydroxyapatite coating than without it [10,39]. A significant decrease in these properties was observed in all tested specimens. The decrease in mechanical values obtained for the samples with hydroxyapatite (for deposition current values of 2.5 and 3.5 mA) confirms that the nanoindentation tests were for the coatings and not for the substrate. 

**Figure 6:**
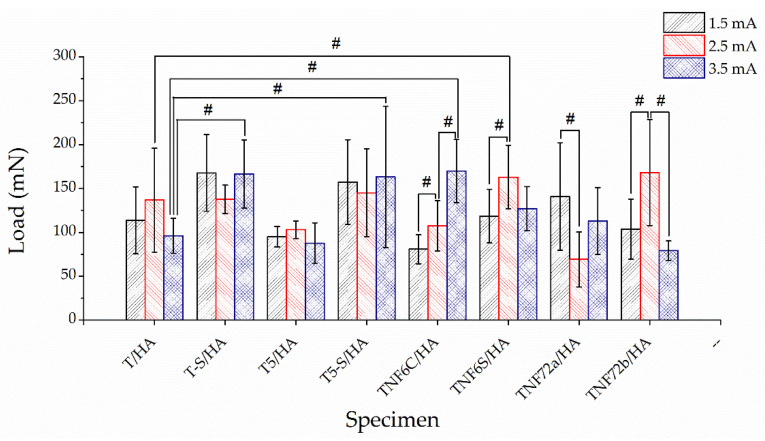
Nanoscratch-test results (adhesion load) of studied: T/HA, T-S/HA, T5/HA, T5-S/HA, TNF6C/HA, TNF6S/HA, TNF72a/HA, and TNF72b/HA samples at various currents (1.5, 2.5, 3.5 mA); (# significantly different according to one-way ANOVA test followed by Bonferroni’s multiple comparison test, *p* < 0.05).

The results of the nanoscratch-test measurements for TS and T5-S samples (Figure S5c) indicate that there is no effect of the modification parameters on its adhesion. Figure 6 shows the results of the nanoscratch-test measurements. The effect of deposition current on adhesion was observed for samples from the TNF6C/HA, TNF6S/HA, TNF72a/HA, and TNF72b/HA groups. However, strict correlations were not observed for these groups: for TNF6C/HA samples, an increase in adhesion was observed with increasing intensity: for TNF6S/HA and TNF72b/HA samples, an initial increase, then a decrease; for TNF72a/HA, an initial decrease, then an increase. Significant differences between HA coatings deposited at the same current value relative to HA coatings deposited on unmodified titanium (specimens T/HA) were obtained for TNF6S/HA (2.5 mA), T-S/HA (3.5 mA), T5-S/HA (3.5 mA), and TNF6C/HA (3.5 mA) specimens. Improved adhesion was observed in all the above. For all tested materials, the best mechanical properties (relative to human cortical bone), while maintaining the highest adhesion value, were characterized by samples from the TNF6C/HA group (3.5 mA). 

## 4. Discussion

The search for optimal conditions of the cathodic electrodeposition of HA on the surface of titanium implants/titanium alloys is one of the crucial issues that research on the design and manufacture of modern implants is focused on [16,30,51,60,61,62]. Electrodeposition is used in surface engineering electrochemical techniques, in which two electrodes (connected to an electrical generator) are immersed in an aqueous solution containing calcium and phosphate ions [16,17,26,63]. It should be noted that besides the deposition process conditions, the substrate’s type and properties directly impact the morphology, structure, and mechanical and biological properties of the produced HA layers. Therefore, our research aimed to select the suitable, universal, and reproducible conditions for the cathodic deposition of HA on the surfaces of Ti6Al4V alloy biomaterials with different surface morphologies and appropriate mechanical properties. 

The electrolyte used during the HA electrodeposition consisted of calcium nitrate tetrahydrate ((CaNO_3_)_2_·4H_2_O) and ammonium dihydrogen phosphate (NH_4_(H_2_PO_4_) [17,51,52,64,65,66,67]. However, in all our experiments, the electrolyte mentioned above was enriched by adding ethylenediaminetetraacetic acid disodium salt (EDTA-2Na). The analysis of the literature data showed that the use of the chelating agent, which is ethylenediaminetetraacetic acid (EDTA), on the properties, morphology, and crystallinity of HA layers deposited on titanium implants has not been fully uncovered so far [47,64,65,68]. According to Zhao et al. [69], the addition of the EDTA-2Na (c = 3.5 · 10^−2^ M) to the electrolyte improved the corrosion resistance and the crystallinity of formed HA layers. It was noted that the increase in the biodegradability of these layers affects the formation of calcium phosphate particles. Morphologically, the resulting HA layers on the magnesium alloy resembled flowers [69]. He et al. [45], studying the effect of EDTA on the deposition of HA coating on Ti6Al4V alloy surface. According to this report, in the absence of a chelating agent, the HA crystals formed were not very dense and had a needle- or rod-like shape, which was consistent with our results. Moreover, no effect of EDTA on changes in the phase composition of the HA layer was found. He et al. proved that increasing EDTA concentration decreases the thickness of the hydroxyapatite layer, which is associated with a decreasing concentration of available calcium ions in the solution. The bonding force between the substrate and the HA layer achieved a maximum value of 16.8 MPa when the EDTA-2Na concentration was 7.5 · 10^−4^ M (the bonding force in this experiments was assessed on the basis of the HA layer breaking strength) [45]. The EDTA-2Na concentration used in our investigations amounted 1.5 · 10^−3^ M. Adhesion force between the substrate and the HA layer (measures using nanoindentation method) varied from 69 mN (TNF72a, I = 2.5 mA) up to 170 mN (TNF6C/HA, I = 3.5mA), depending on the type of surface modification. This issue will be discussed in a later part of this paper. Court et al. [58] pointed out that the electrodeposition temperature also affects the morphology and structure of hydroxyapatite layers on titanium substrates. The HA layers revealed homogenous plate-like structures. However, deposition at 348 K led to denser coatings, covering the whole surface with a more compact layer than in deposition at 323 K. At lower temperatures, the deposition rate of the HA layer was lower, and the resulting layers were thinner. HA layers deposited at 348 K were more hydrophilic (contact angle 27.4°) than those deposited at 323 K (contact angle 76.1°) [58]. In our experiments, hydroxyapatite was deposited on modified titanium alloy surfaces of different morphology at 338 K as an optimal temperature. The analysis of the XRD patterns of studied samples revealed that the intensity of peaks assigned to HA layers was the greatest. Moreover, all produced hydroxyapatite layers showed superhydrophilic properties, which is highly desirable for biomedical applications [23,58,59,70].

It is assumed that the formation of hydrophilic coatings promotes apatite growth due to the increased ion exchange capacity from the SBF solution [42,44]. This effect is consistent with our earlier investigations on apatite growth on intermediate TiO_2_ and titanate coatings. The apatite growth from SBF solution was noticed on amphiphilic titanate substrates, but it was not observed for the hydrophobic TiO_2_ nanoporous surfaces [38]. Superhydrophilic surfaces exhibit increased surface tension and a high potential to form hydrated layers with surrounding water molecules through the hydrogen bonds [23,60,61,62,71,72]. He et al. [45], studying the issue of the substrate influence on the formation of the HA layer, used the electrodeposition method on a pre-treated alkali-sodium Ti6Al4V substrate. They showed that by varying the current density values between 1.25 and 3.61 mA/cm^2^, no differences in the composition and grain size of the hydroxyapatite layer are observed. However, as the current density increases, the thickness of the HA layers increases and the crystal structure changes from porous to dense (1.25–2.5 mA/cm^2^) and back to porous (2.5–3.61 mA/cm^2^) [45]. Meanwhile Gu et al. [63] obtained the most homogeneous HA layers using lower current densities, i.e., 5 mA/cm^2^. They observed that the higher the current density, the more heterogeneous the layer. At a current density of 10 mA/cm^2^, they obtained a non-uniform coating of seaweed-like crystals with different grain sizes [63]. In contrast, Chakraborty et al. [64] exhibited that the 10 mA/cm^2^ as the most optimal current density. The hydroxyapatite layers on SS316 substrate obtained at this value are porous, crystallite sizes in the nano range [64]. There are also reports that an increase in current density from 10 to 20 mA/cm^2^ leads to an increased volume fraction of microcracks, changing surface cracks to cracks formed along the deposited layer’s cross-sections [41,65,66,67,68]. 

Searching for the optimal conditions of the cathodic electrodeposition of HA layers on Ti6Al4V substrates, we decided to study the influence of current densities (input: J = 7.5 mA/cm^2^ (I = 1.5 mA), J = 12.5 mA/cm^2^ (I = 2.5 mA), and J = 17.5 mA/cm^2^ (I= 3.5 mA)) on the physicochemical and mechanical properties of the produced coatings. Our results were compatible with most earlier reports concerning the HA layer deposition on the pure titanium alloy. A current density of 17.5 mA/cm^2^ lead to numerous micro-cracks, delaminating the HA layer from the substrate. It can be attributed to the increased cathodic hydrogen evolution reaction (released with increasing current density), leading to the cracking and the porous structure formation of the HA layer [21,45,73,74]. However, we observe that the influence of the type of surface modification used on the Ti6Al4V alloy substrate has a significant effect on the hydroxyapatite layers formed. The applied current I = 1.5 mA (J = 7.5 mA/cm^2^) is too low for the formation of hydroxyapatite crystals in the case of hydroxyapatite deposition on T5 porous or TNF6 nanofibrous coating. On the surfaces of pre-treated sodium-alkali samples, i.e., T-S and T5-S HA, hydroxyapatite forms nanoplates dispersed over the coating surface. Whereas on the surface of nanofibrous TNF72 we observe the formation of HA in the form of flower-like (TNF72a) or nanoplatelets (TNF72b). We observed no visible changes in the morphology of hydroxyapatite crystals between the applied current density value of 12.5 mA/cm^2^ (I = 2.5 mA) and 17.5 mA/cm^2^ (I = 3.5 mA). For all tested substrates, the hydroxyapatite layers show a floral morphology composed of numerous nanoplatelets. When current density increases, the change of HA crystalline structure from dispersed to densely packed is visible, and the thickness of HA layers grows. The modification of the titanium substrate had no significant effect on the thickness of the HA layers obtained when the current density was 12.5 mA/cm^2^. The obtained layer thicknesses were between 11.61 µm (TNF6C) and 12.90 µm (TNF72a). The exception at 2.5 and 3.5 mA was the TNF72b sample, for which the layer obtained was the thickest at 14.51 and 20.8 µm, respectively. This layer also exhibited the weakest mechanical properties. At a current intensity of 3.5 mA, we noted wider differences in layer thicknesses depending on the coating on which the hydroxyapatite was deposited. The obtained layer thicknesses ranged from 13.60 µm (TNF6C) to 17.60 µm (T-S).

In order to determine which of the Ti6Al4V surface modifications is most desirable for the deposition of hydroxyapatite layers, mechanical studies were crucial. Improving the mechanical strength of hydroxyapatite layers has been a challenge for many years. The interfacial adhesion of HA to the substrate is weak [21,30,43,75,76,77]. In our research, to increase the adhesion between the substrate and the hydroxyapatite layer, we used intermediate coatings in the form of nanotubes (T5), nanofibres (TNF), and titanates (T-S, T5-S). We had previously analyzed the chosen nanocoatings in depth for their physicochemical, mechanical, and biointegrative properties [10,11,38,39,40]. Integrating them into the HA layer brings many benefits related to increasing the implant’s bioactivity and improving the HA layers’ mechanical properties. The obtained HA layers were characterized in terms of adhesion, hardness, and Young’s modulus. In order to avoid the shielding effect, which is associated with a significant loss of bone mass, it is aimed that Young’s modulus of the implants is close to the elastic modulus of cortical bone (about 20 GPa) [78,79]. 

The previous reports suggest that the increased interfacial adhesion strength between the titanium implant and the apatite layer is due to the roughness of the biomaterial surface [80,81,82,83]. It is thought that a larger surface area and physical blockage are generated between the nanotube coating and the apatite layer [80,81,82,83]. Our research confirmed that the substrate’s surface roughness affects the hydroxyapatite layer’s obtained mechanical properties. Our produced intermediate oxide coatings show high surface roughness parameters (S_a_) compared to the unmodified titanium alloy [10,39]. We observed the highest value of the S_a_ parameter for the TNF6S intermediate nanofibrous coating (0.30 µm) and the lowest value for the TNF72a nanofibrous coating (0.06 µm) [39]. Using the cathodic HA deposition technique on intermediate coatings, we did not observe the delamination of the layer. In contrast, delamination was evident when depositing the HA layer directly onto a Ti6Al4V substrate (low value S_a_ = 0.02 µm) [10]. With the use of nanoporous (T5) titanate (T5-S and T5) coatings as interlayer systems, the dense HA layers with optimal mechanical properties were produced using a current of 2.5 mA. Young’s modulus for the samples mentioned above was close to that of cortical bone and was 35.58 GPa for T5/HA, 28.05 GPa for T-S/HA, and 19.50 GPa for T5-S/HA, respectively. Among the materials as mentioned earlier, the adhesion of HA layers was higher for the T-S (137.62 mN) and T5-S (145.22 mN) titanate coatings in comparison to T5/HA (103.11 mN) nanoporous coatings. This may be related to the formation of a bonding layer, i.e., CaTiO_3_ (chemical bonding) [43,84,85]. According to our previous studies, the Na_2_Ti_3_O_7_ layer supports spontaneous apatite deposition, as it is capable of actively exchanging calcium cations in a simulated SBF body fluid solution, thus converting to calcium titanate. The T5-S layer formed by the alkali-sodium treatment of the T5 nanoporous coating shows faster apatite growth in SBF solution than the T-S layer formed by the alkali-sodium treatment of pure titanium alloy [38]. In addition, the rough surface resulting from the alkaline pretreatment increases the bonding strength [84,85,86,87]. Blackwood et al. [88] showed that the crystallinity and adhesion of HA could be improved by the sodium-alkali pretreatment of titanium. However, they suggested it is also crucial to additionally apply a heat treatment at 873 K or add H_2_O_2_ to the electrolyte [88]. Ahmadi et al. [25] reported that the production of a nanotube coating in the anodization process can significantly increase the adhesion strength of hydroxyapatite to the substrate. The bond strength for the HA/Ti6Al4V system was 12.8 ± 2 MPa, while for the HA/TiO_2_ nanotube nanocomposite it was 23.1 ± 4 MPa. The formation of a rough surface at the HA/TiO_2_ nanotube interface strengthens the cohesion forces. As a result of filling the voids, a compact HA/TiO_2_ layer is formed. It can also be seen that titanium dioxide nanoparticles in the HA layer are able to relax residual stresses, which are usually responsible for microcracks in the hydroxyapatite deposition process [25,89,90]. Besides the anodization process, the sodium-alkali treatment, a variety of etchants, such as HNO_3_, H_2_SO_4_, HCl, and HF, are commonly used to pretreat the substrate. Using the compounds mentioned above in concentrations between 50 and 75% improved the adhesion strength at the coating/implant interface [21,91,92,93,94,95]. As an intermediate coating between the titanium alloy substrate and the HA coating, we also used TiO_2_ nanofibres (TNF). These studies revealed that some samples were chemically etched in a mixture of concentrated HCl and H_2_O and then heated in a 30% H_2_O_2_ solution at 358 K for 6 h. Depending on the heating procedure, the samples were designated as TNF6S (heating in an incubator) and TNF6C (heating under a reflux condenser). Another sample was etched, respectively, in (a) 2 M HF solution for 10 s (TNF72a), (b) in a 1:4:5 mixture of HF:HNO_3_:H_2_O for 30 s (TNF72b), and then heated in 30 wt.% H_2_O_2_ solution at 358 K for 72h. The obtained TNF samples are amorphous and contain rutile (TNF72) or anatase (TNF6) phase domains [39,40]. In contrast to nanoporous and titanate samples, we noticed that the optimum mechanical properties of electrodeposition of HA layers on nanofibrous coatings are obtained at 3.5 mA (except TNF72b). Generally, the TNF72b/HA sample at both 2.5 mA and 3.5 mA exhibits the weakest hardness (0.13 and 0.17 GPa, respectively) and a very low Young’s modulus (6.81 GPa and 2.98 GPa, respectively) compared to other biomaterials. Surprisingly, the adhesion strength of the HA layers to the TNF72b coating is high at 2.5 mA and is 168.25 mN. As the current increases to 3.5 mA, it rapidly decreases to 79.25 mN. It possibly can be related to a too-thick deposited HA layer on the TNF72b coating. In this case, the deposition of the thickest HA layer (20.8 um) was noticed. The TNF6C/HA sample exhibits the best mechanical properties of all analyzed samples, where the current intensity was 3.5 mA. The thickness of the resulting HA layer is 13.60 um. The Young’s modulus is close to that of cortical bone and was 25.96 GPa, while the adhesion force is 169.93 mN. So far, there are no reports on the electrodeposition of the hydroxyapatite on nanofibrous Ti6Al4V substrates.

Robertson et al. [96] presented a different approach to this issue and used a sol-gel method to deposit hydroxyapatite on a Ti6Al4V–ELI substrate, obtaining the layer with a thickness of 73.3 um. They also tested anodized Ti6Al4V with an HA layer, for which the thickness increased to 84.97 um. However, it should be noted that the main function of the oxide intermediate coatings is to improve the adhesion of the ceramic hydroxyapatite layer to the titanium substrate, so the smaller the thickness of the HA layer, the more chance of avoiding its delamination and introducing additional stresses. In the method we used, the thickness of the HA layers obtained varied between 9.67 µm (T/HA I = 2.5mA) and 20.80 µm (TNF72b I = 3.5 mA), depending on the intermediate coating used and the applied current. An additional advantage of using the cathodic deposition method is that the deposition and sintering time of the hydroxyapatite layer is significantly shorter, and the ageing step (necessary in the case of the sol-gel method) is omitted, which increases the chances of using this method in industry [97,98]. Commercially, the most commonly used method for the deposition of hydroxyapatite coatings is plasma spraying. The resulting layers typically achieve thicknesses in the range of 20–50 µm [99]. However, the limitations of this method are mainly the high energy and temperature of the plasma, which can cause rapid thermal decomposition of HA and numerous residual stresses [100]. Singh et al. showed that in plasma-sprayed coatings, the hydroxyapatite particles on the Ti6Al4V alloy melt completely or partially due to the high plasma temperature. The researchers obtained a very high surface roughness value (R_a_ = 5.43 µm) for the Ti6Al4V/HA systems [37]. The R_a_ value obtained is nearly ten times higher than that achieved in our study (0.57 µm for T/HA). Plasma-sprayed HA coatings have a much rougher surface compared to HA coatings produced by cathodic deposition or sol-gel [37,96]. By applying plasma spraying with induction preheating (400–600 °C), Fomin et al. [101] revealed that the resulting HA layers on Ti implants exhibited a stoichiometric-like chemical composition, a homogeneous nanostructure, and morphologically resembled micro-sized splats with numerous nanograins. Moreover, the HA layers on Ti substrates showed high hardness in the range of 0.9–12 GPa and elastic modulus in the range of 7–16 GPa. Unfortunately, during this work, the adhesion strength between the coating and the substrate was not determined. The results of our research revealed that the morphology of the produced HA coatings was more floral, with numerous nanoplatelets. Moreover, the produced HA layers exhibited close mechanical parameters to cortical bone (H = 0.29 GPa, Young’s modulus = 25.96 GPa) and high adhesion force = 169.93 mN for the TNF6C/HA sample. Ritwik et al. [102] produced a thin layer (30 µm) of hydroxyapatite on a Ti6Al4V substrate using a dip-coating process. They used shellac (a natural resin) as an intermediate coating. The researchers showed that the deposition of a shellac layer between the HA and the Ti6Al4V substrate increases the adhesion force from 1.08 MPa (Ti6Al4V/HA) to 7.14 MPa (Ti6Al4V/Shellac/HA). The average surface roughness (R_a_) values measured were 0.93 µm [102]. The cathodic deposition of hydroxyapatite, which we optimized, allowed us to obtain thinner hydroxyapatite layers (from 10 µm to 20 µm, depending on the type of intermediate coating used) and two to three times lower roughness.

## 5. Conclusions

In order to increase the adhesion strength of hydroxyapatite layers (HA) to Ti6Al4V alloy substrates, we researched the morphology, structure, and mechanical properties of HA layers deposited by the cathodic electrodeposition method. The results proved that the above coatings’ parameters depend not only on temperature, time, current density, and the addition of EDTA-2Na to the electrolyte solution but also on the method of the pre-modification of the alloy surface. The fabrication of an intermediate layer between Ti6Al4V substrates surface and hydroxyapatite coating, which could be the TiO_2_ nanoporous layer (T5), TiO_2_ nanofiber (TNF6, TNF72), and titanate (T5-S, T-S), significantly improve the mechanical properties of the HA layers. Our investigations revealed that Ti6Al4V/TNF6C system (i.e., TiO_2_ nanofibrous coatings formed by chemical oxidation in a 30% H_2_O_2_ solution at 358 K under a reflux condenser) is the most appropriate for the fabrication of implants covered with a mechanical permanent hydroxyapatite layer. However, the obtained physicochemical and mechanical parameters are also satisfactory for the other intermediate layers, i.e., T5, T5-S, T-S, and TNF6S with a hydroxyapatite layer. Double-layer coatings (TNF6C/HA, T5/HA, T-S/HA, T5-S/HA, and TNF6S/HA) offer very promising results (the mechanical properties like human cortical bone), which can be used in the construction of modern orthopedic or dental implants. 

In the next step of our research, in vitro investigations of the biomaterials mentioned above will be conducted to confirm their potential medical applications. We will focus on Ti6Al4V/intermediate layer/HA systems, investigating their effects on the survival and proliferation of human MG-63 osteoblasts-like, mouse L929 fibroblast, and adipose-derived human mesenchymal stem cell (ADSC) cultures seeded on their surface in vitro. We will also determine the antimicrobial activity of the produced systems. Results on the apatite-forming ability of coatings immersed in simulated body fluid (SBF) will be presented.

## Figures and Tables

**Table 1:** Ca/P ratios obtained from EDS measurements for the samples with hydroxyapatite layers at different currents.

Ca/P (Mole Ratio) of HA Layer at Different Currents			
Sample	I = 1.5 mA	I = 2.5 mA	I = 3.5 mA
T/HA	1.83	1.60	1.54
T-S/HA	1.45	1.69	1.73
T5/HA	0.82	1.58	1.82
T5-S/HA	1.39	1.62	1.92
TNF6c/HA	1.13	1.45	1.76
TNF6s/HA	1.66	1.54	1.58
TNF72a/HA	1.70	1.96	1.75
TNF72b/HA	1.58	1.63	1.65

**Table 2:** DRIFT frequencies and corresponding functional groups in hydroxyapatite samples.

Functional Groups	Frequencies (Experimental) (cm^−^^1^)	Frequencies (Reference) (cm^−^^1^)	Reference
ν_3_(PO_4_^3−^)	1188–1006	1200–960	[43,44,45,46,47,48,49,50]
ν_1_(PO_4_^3−^)	960–956	963–960	[43,44,47,48,49,51]
ν_4_(PO_4_^3−^)	601–531	660–520	[19,43,44,45,46,47,48,50,51,52,53]
ν_1_(CO_3_^2−^)	1460–1398	1500–1400	[43,44,45,46,47,48,49,51,52]
ν_3_ or ν_4_(CO_3_^2−^)	875–867	880–865	[43,44,45,49,50,54]
ν_3_(CO_3_^2−^)	1323	1300, 1321	[47,48]
ν(OH)	3456–3000	3571–3420	[41,42,43,44,45,46,49,50,51,55]
σ(OH)	1653–1594	1650–1631	[43,44,45,50,51,55]

**Table 3:** Changes of the roughness value (R_a_) (µm) of the HA layer deposited at different currents and the surface of different substrates. The values are expressed as means ± SEM of five independent experiments.

	I = 2.5 mA	I = 3.5 mA
Sample	Roughness (R_a_) (µm)	
T/HA	0.57 ± 0.07	0.33 ± 0.00
T-S/HA	0.21 ± 0.03	0.29 ± 0.04
T5/HA	0.21 ± 0.00	0.31 ± 0.02
T5-S/HA	0.20 ± 0.05	0.38 ± 0.05
TNF6C/HA	0.20 ± 0.01	0.61 ± 0.03
TNF6S/HA	0.15 ± 0.00	0.43 ± 0.03
TNF72a/HA	0.13 ± 0.01	0.32 ± 0.02
TNF72b/HA	0.11 ± 0.01	0.13 ± 0.00

## Data Availability

Data sharing is not applicable in this article.

## Supplementary Materials

The following supporting information can be downloaded at: https://www.mdpi.com/article/10.3390/ma15196925/s1; Figure S1: SEM cross-section images of the T/HA (a), T-S/HA (b), T5/HA (c), T5-S/HA (d), TNF6C/HA (e), TNF6S/HA (f), TNF72a/HA (g), and TNF72b/HA (h) samples at 2.5 mA current.; Figure S2: SEM cross-section images of the T/HA (a), T-S/HA (b), T5/HA (c), T5-S/HA (d), TNF6C/HA (e), TNF6S/HA (f), TNF72a/HA (g), and TNF72b/HA (h) samples at 3.5 mA current; Figure S3: Energy-dispersive spectroscopy (EDS) spectra and quantitative data of the T5-S samples with hydroxyapatite layers at various currents: 1.5 mA (a), 2.5 mA (b), and 3.5 mA (c); Figure S4: DRIFT spectra of studied T/HA (a), T-S/HA (b), T5/HA (c), T5-S/HA (d), TNF6C/HA (e), TNF6S/HA (f), TNF72a/HA (g), and TNF72b/HA (h) samples at various currents (1.5, 2.5, 3.5 mA); Figure S5: Nanomechanical properties (hardness—left and Young’s modulus—right) of T-S and T5-S specimens.
