# Supplementary figures and images for: Evaluation of the Cathodic Electrodeposition Effectiveness of the Hydroxyapatite Layer Used in Surface Modification of Ti6Al4V-Based Biomaterials
Source: Materials (Basel). 2022 Oct 6;15(19):6925. doi: 10.3390/ma15196925 (PMC9572782; doi:10.3390/ma15196925)

## Slide 1
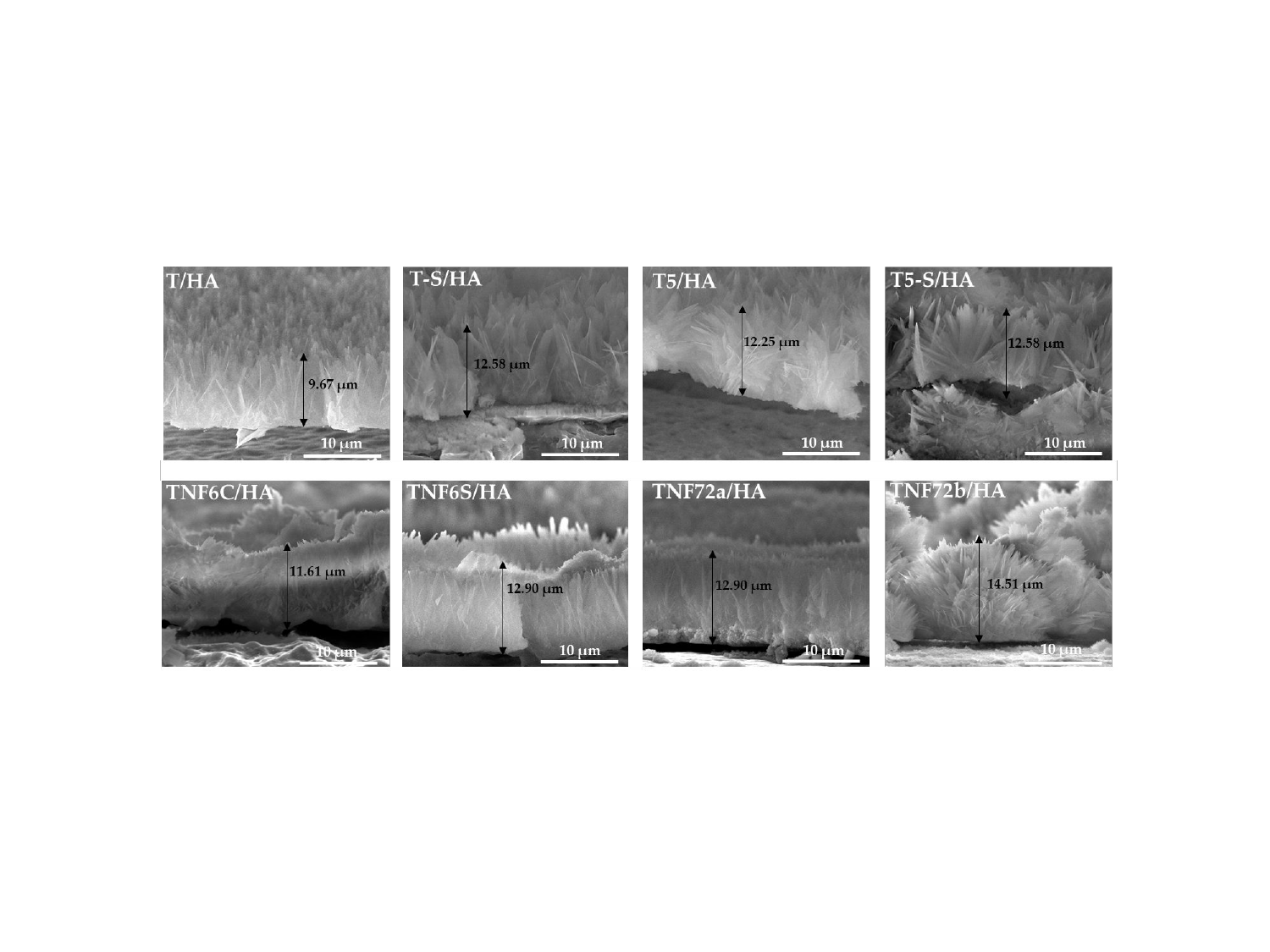

Supplement: Supplementary file 1 [file materials-15-06925-s001.zip › Figure S1.pptx]

## Slide 1
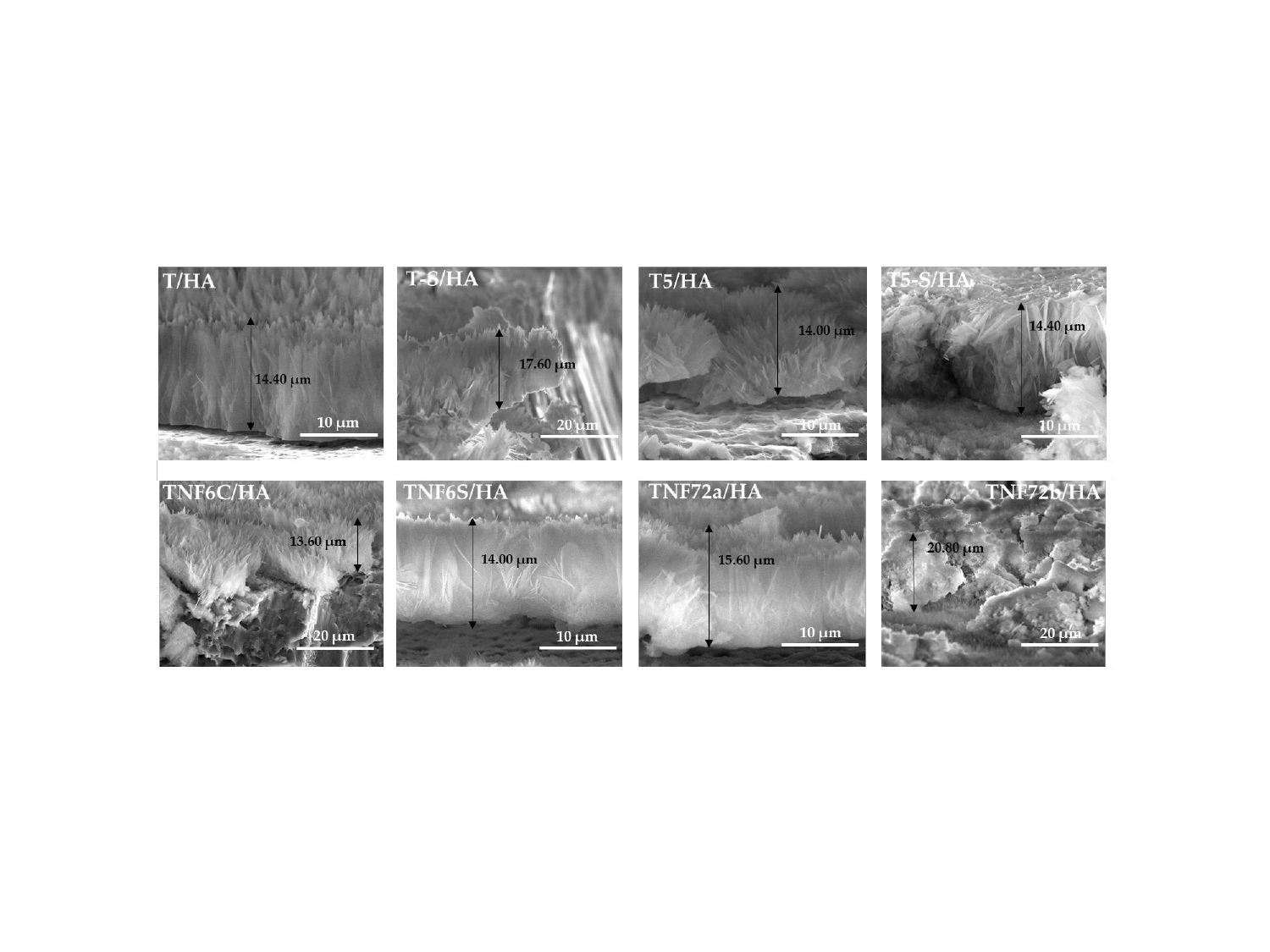

Supplement: Supplementary file 1 [file materials-15-06925-s001.zip › Figure S2.pptx]

## Slide 1
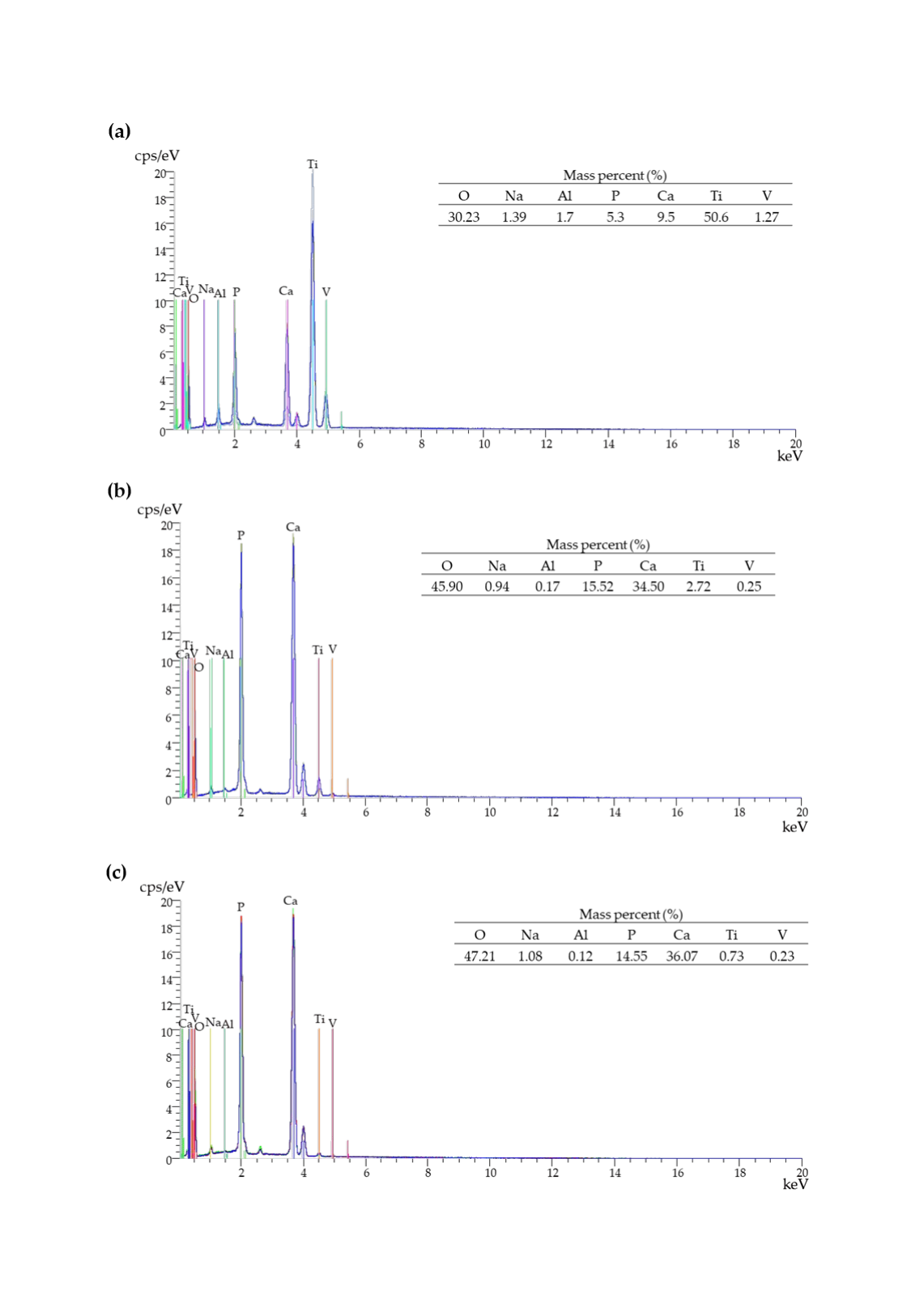

Supplement: Supplementary file 1 [file materials-15-06925-s001.zip › Figure S3.pptx]

## Slide 1
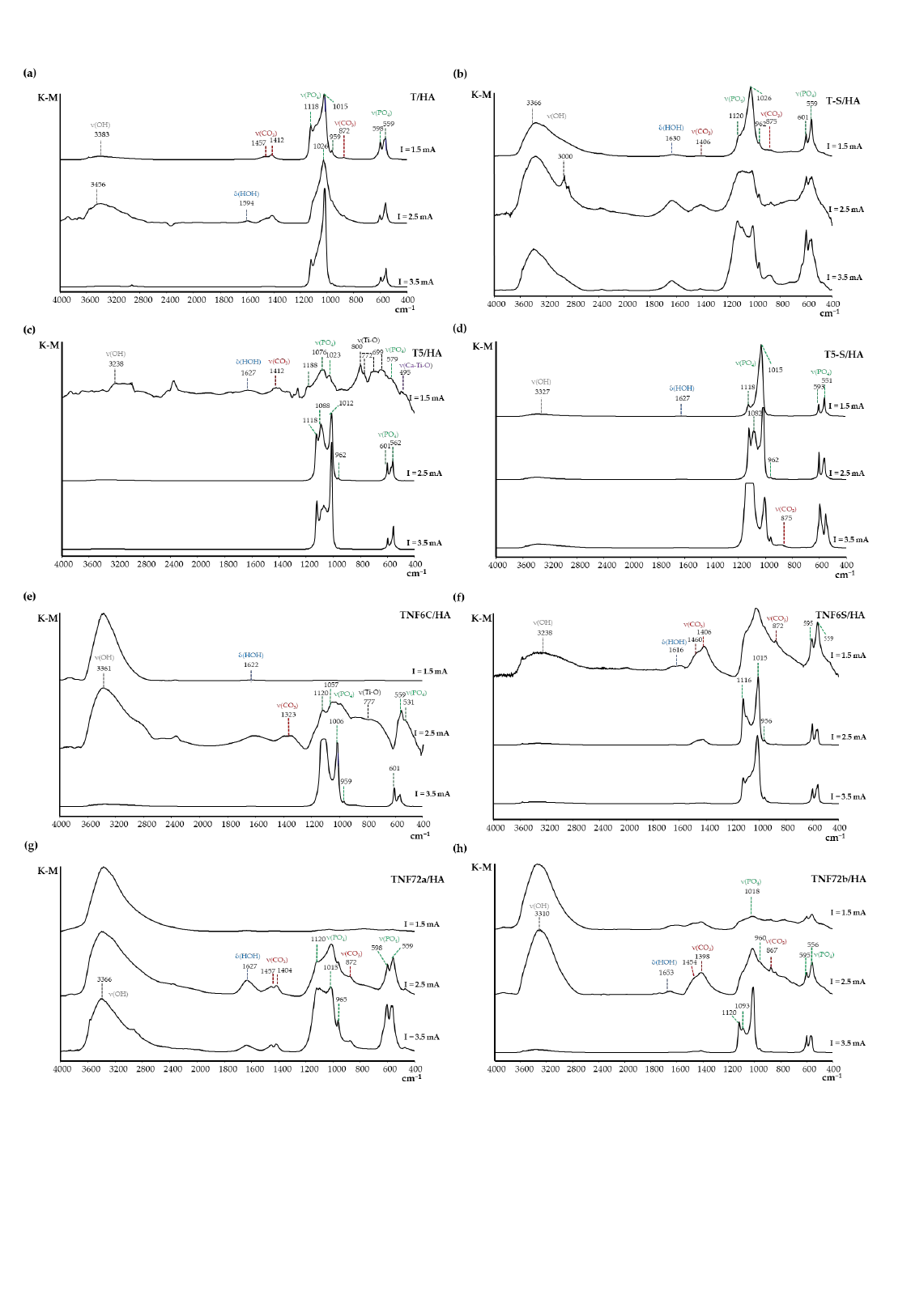

Supplement: Supplementary file 1 [file materials-15-06925-s001.zip › Figure S4.pptx]

## Slide 1
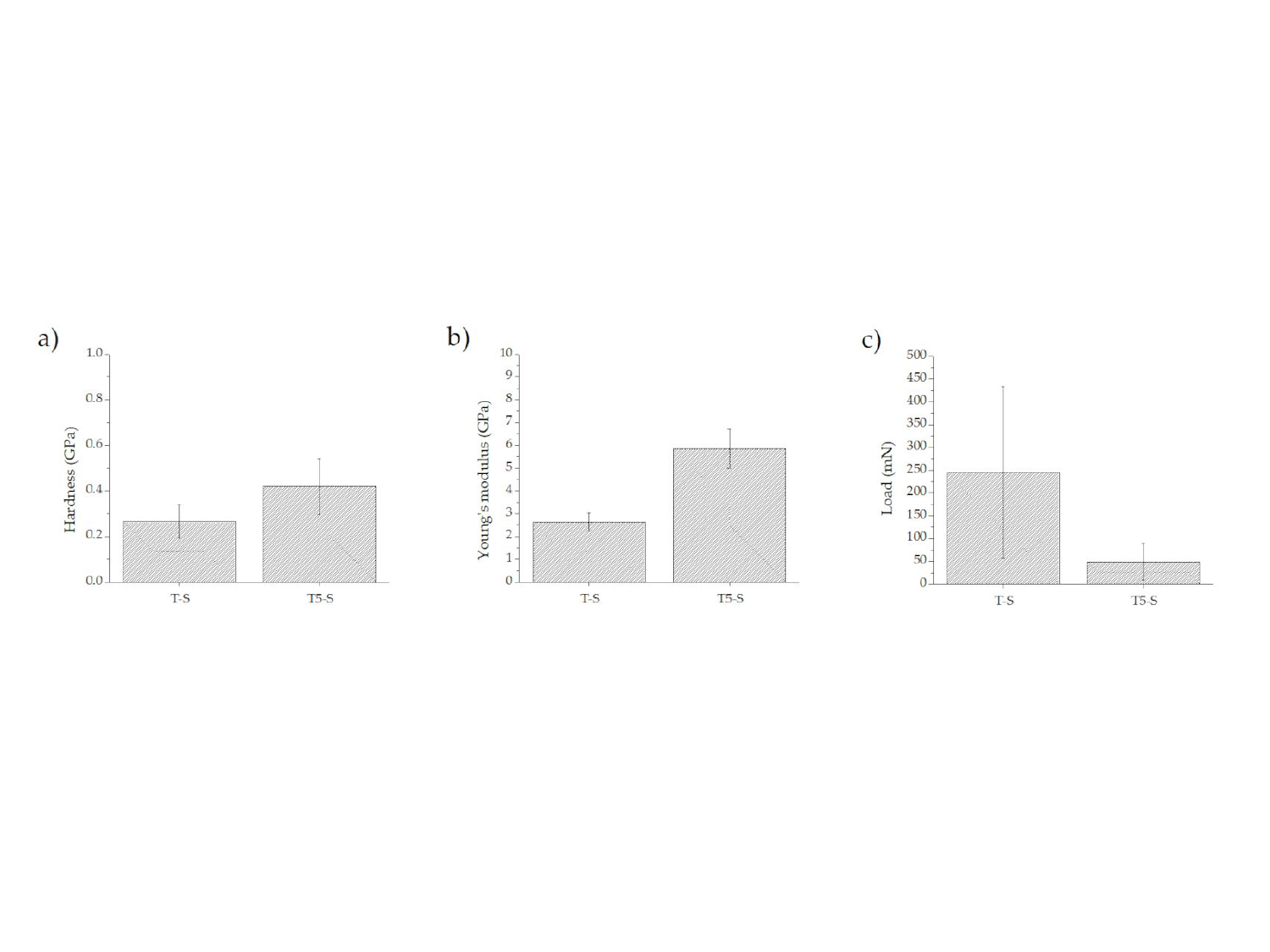

Supplement: Supplementary file 1 [file materials-15-06925-s001.zip › Figure S5.pptx]
